# An Epidemiological Study of COVID-19 Cases in Al-Leith, Saudi Arabia

**DOI:** 10.7759/cureus.20457

**Published:** 2021-12-16

**Authors:** Mohammed Elawad, Dalal Al-Thubyani, Abrar Almhdawi, Elaf Al Brakati, Fatimah Al Hatami, Omar B Ahmed

**Affiliations:** 1 Public Health, Faculty of Health Sciences, Umm Al-Qura University, Al-Leith, SAU; 2 Public Health, Umm Al-Qura University, Al-Leith, SAU; 3 Department of Environmental and Health Research, Umm Al-Qura University, Makkah, SAU

**Keywords:** coronavirus, precautions, epidemiological, al-lieth, covid-19

## Abstract

Background and objective

Ever since the severe acute respiratory syndrome coronavirus 2 (SARS-CoV-2) was first detected in December 2019, more than 0.550 million cases of coronavirus disease 2019 (COVID-19) have been reported in the kingdom of Saudi Arabia (KSA) and the number is on the rise. In this study, we aimed to perform an epidemiological analysis of COVID-19 cases in Al-Leith, KSA.

Methods

A community-based descriptive study was carried out to assess the epidemiology of COVID-19 in Al-Leith, KSA. The relevant data were collected using a questionnaire designed for this study, which included questions on basic information and characteristics in addition to data on COVID-19. Data were analyzed using the SPSS Statistics software (IBM, Armonk, NY).

Results

The significant findings were as follows: people aged between 26-40 years were more affected (55.7%) than other age groups, and more than half (74.1%) of COVID-19 patients were female; most of them were employed (85, 48,9%), and most of those got infected through direct contact (137, 78.7%) with other infected people. About 163 (93.7%) cases were asymptomatic, and 168 (96.6%) cases were notified to the health authorities. The findings also illustrated that 78 (44.8%) COVID-19 cases suffered from psychological impact due to infection; 83 (47.7%) cases had at least one case in the family before they became infected. The majority of cases (93.7%) suffered from symptomatic COVID-19. A considerable number of COVID-19 patients did not follow precautions before and after infection.

Conclusions

The study concluded that various age groups were susceptible to developing COVID-19, and direct contact was the main mode of transmission. Moreover, a considerable number of infected people did not adhere to precautionary measures.

## Introduction

The severe acute respiratory syndrome coronavirus 2 (SARS-CoV-2), which causes coronavirus disease 2019 (COVID-19), was first detected in December 2019 in Wuhan, China [1]. Since then, It has quickly spread to other countries and continents, and the World Health Organization (WHO) declared it a pandemic on March 11, 2020. Approximately 260 million cases have been reported worldwide as of November 2021 [2], including approximately 0.550 million in the kingdom of Saudi Arabia (KSA) [3]. The most common symptoms of the COVID-19 disease are fever, cough, sore throat, and shortness of breath. The statistics related to the incidence and mortality for COVID-19 have varied widely worldwide [3]. Cumulative incidence rates as of May 31, 2021, ranged from 0.3 to 17,700 per 100,000 population, while COVID-19-associated mortality rates during the same period ranged between 0.03 and 308 casualties per 100,000 population worldwide [4]. Case fatality rates (CFR) have been estimated to range from 0.8 to 15.2% [5,6].

The authorities in the Saudi government have implemented many important preventive measures to mitigate the spread of COVID-19. Older people, especially those with chronic health conditions, are at the highest risk of morbidity and mortality related to COVID-19 [5,6]. While both genders are susceptible to COVID-19 infection, some reports have shown that men have higher mortality and morbidity rates than women [7], while other data have shown variable mortality and morbidity rates among both genders; however, these rates may vary from country to country and between regions within a country [8]. In addition, the mortality rate for both sexes may increase with the increase in age, particularly in people aged older than 30 years, where males show a higher risk of mortality than females [9,10]. Worldwide, the male-to-female ratio is above 1 and may reach higher than 2 in some countries such as Thailand, the Netherlands, Albania, and Costa Rica [9]. Children also may be infected, particularly in the upper respiratory tract than the lower respiratory tract, and may recover within two weeks [11,12]. Early reports of the COVID-19 outbreak indicated that patients suffering from chronic diseases such as diabetes, cardiovascular diseases, chronic kidney disease, and chronic respiratory diseases may be affected by a severe form of COVID-19 [13,14]. Although many studies have reported low rates of COVID-19 incidence in asthmatic patients, it has been shown that asthma may be underreported in COVID-19 patients [15,16]. In addition, COVID-19 disease has been reported to be significantly severe in patients with cardiovascular diseases, with a 5-10-fold elevated risk of mortality compared to others who are infected [17,18]. In light of these factors, we conducted a community-based descriptive study to examine the epidemiology of COVID-19 in Al-Leith, KSA.

## Materials and methods

We employed a descriptive community-based study design. The study was conducted in the year 2020. Al-Leith is a city in the Tihamah region and lies southwest of the holy city of Makkah and 190 km south of Jeddah in the KSA. It is the fifth most populous city in the Makkah Province and one of the large seaports in the KSA on the Red Sea. The estimated population of Al-Leith is over 72,000 people. The city has general hospitals and primary healthcare centers that are equipped to treat patients with COVID-19. The study population comprised COVID-19 patients who were residents of the Al-Leith town, where 656 cases were reported in 2020. The inclusion criteria were as follows: any COVID-19 patient who resided in Al-Leith. Although it was determined that about 10% of the total cases in Al-Leith would be sufficient for our analysis, we recruited more people than that to enrich and strengthen the findings, and 174 participants were included in the final analysis. The participants were selected by simple random sampling. The size of the sample was determined according to the response of participants. The relevant data were collected using a questionnaire designed for this study, which included questions on basic information and characteristics in addition to data on COVID-19. The collected data were analyzed using the SPSS Statistics software (IBM, Armonk, NY) and the results are shown in tables.

## Results

Table 1 illustrates the distribution of COVID-19 patients in Al-Leith, KSA by age groups; those aged between 26-40 years were affected at a higher rate (55.7%) than other age groups. Table 2 shows the gender-wise distribution of COVID-19 patients in Al-Leith; about 74.1% of COVID-19 patients were female. Table 3 displays the occupation-wise distribution of COVID-19 patients in Al-Leith. It was observed that most of them were employed (85, 48,9%). Table 4 highlights the mode of infection of COVID-19 patients in Al-Leith. We found that most patients became infected through direct contact with those already infected (137, 78.7%).

**Table 1:** Age-wise distribution of COVID-19 patients in Al-Leith, Saudi Arabia (n=174)

		Frequency	%
Age in years	6-15	1	0.6
	16-25	50	28.7
	26-40	97	55.7
	41-64	25	14.4
	≥65	1	0.6
	Total	174	100

**Table 2:** Gender-wise distribution of COVID-19 patients in Al-Leith, Saudi Arabia (n=174)

		Frequency	%
Gender	Male	45	25.9
	Female	129	74.1
	Total	174	100

**Table 3:** Occupation-wise distribution of COVID-19 patients in Al-Leith, Saudi Arabia (n=174)

		Frequency	%
Occupation	Student	39	22.4
	Employee	85	48.9
	Worker	4	2.3
	Own business	3	1.7
	Unemployed	43	24.7
	Total	174	100

**Table 4:** Method of infection among COVID-19 patients in Al-Leith, Saudi Arabia (n=174)

		Frequency	%
Method of infection	Direct contact	137	78.7
	Hand contamination	10	5.7
	Other	27	15.5
	Total	174	100

Regarding the symptoms of COVID-19 among patients in Al-Leith, most of the cases were symptomatic (93.7%, Table 5). Table 6 shows the data related to COVID-19 patients informing their relatives and friends about their infection; the majority of the infected people (168, 96.6%) reported informing their relatives and friends. Table 7 displays the isolation details of COVID-19 patients in Al-Leith; 168 (96.6%) of the infected people were isolated. Table 8 presents the treatment-related data of COVID-19 patients in Al-Leith; more than 100 people have undergone treatment (123, 70.7%).

**Table 5:** Symptom-related data of COVID-19 patients in Al-Leith, Saudi Arabia (n=174)

		Frequency	%
Symptoms	Symptomatic	163	93.7
	Asymptomatic	11	6.3
	Total	174	100

**Table 6:** Informing the relatives of COVID-19 patients in Al-Leith, Saudi Arabia (n=174)

		Frequency	%
Notification to relatives and friends	Yes	168	96.6
	No	6	3.4
	Total	174	100

**Table 7:** Isolation-related data of COVID-19 patients in Al-Leith, Saudi Arabia (n=174)

		Frequency	%
Isolation	Yes	168	96.6
	No	6	3.4
	Total	174	100

**Table 8:** Treatment of COVID-19 patients in Al-Leith, Saudi Arabia (n=174)

		Frequency	%
Treatment received	Yes	123	70.7
	No	51	29.3
	Total	174	100

Table 9 provides the data related to the psychological impact among COVID-19 patients in Al-Leith; 96 (55.2%) patients were not psychologically affected by COVID-19 and the rest were affected. Table 10 lays out information related to the precautionary measures taken by the COVID-19 patients in Al-Leith before they were infected; 12 (6.9%) patients reported not adhering to any precautionary measures. Table 11 shows the distribution of the cases in the family before the infection of COVID-19 among patients in Al-Leith; 47.7% of patients were infected with COVID-19 from their family members. Table 12 shows the correlation between the age of COVID-19 patients and the presence of clinical symptoms. Patients who suffered from COVID-19 symptoms were predominantly in the age group of 26-40 years, followed by those in the age group 16-25 years.

**Table 9:** Psychological impact among COVID-19 patients in Al-Leith, Saudi Arabia (n=174)

		Frequency	%
Psychological impact	Yes	78	44.8
	No	96	55.2
	Total	174	100

**Table 10:** Precautionary measures before infection among COVID-19 patients in Al-Leith, Saudi Arabia (n=174)

		Frequency	%
Precautionary measures before the infection	Yes	128	73.6
	No	12	6.9
	Occasionally	34	19.5
	Total	174	100

**Table 11:** Cases in the family before infection among COVID-19 patients in Al-Leith, Saudi Arabia (n=174)

		Frequency	%
Cases in the family before the infection	Yes	83	47.7
	No	91	52.3
	Total	174	100

**Table 12:** Correlation between age of COVID-19 patients and the presence of clinical symptoms in Al-Leith, Saudi Arabia (n=174)

		Clinical symptoms		Total
		No	Yes	
Age of patients (years)	6-15	0 (0.0%)	1 (100.0%)	1 (0.57%)
	16-25	3 (6.0%)	47 (94.0%)	50 (28.7%)
	26-40	4 (4.1%)	93 (95.9%)	97 (55.7%)
	41-64	4 (16.0%)	21 (84.0%)	25 (14.4%)
	≥65	0 (0.0%)	1 (100.0%)	1 (0.57%)
Total		11 (6.3%)	163 (93.7%)	174 (100%)

## Discussion

The present study was carried out to assess the epidemiology of COVID-19 in Al-Leith, KSA. The study showed that more than half (74.1%) of COVID-19 patients were females. In contrast with this finding, some reports have shown that men have higher incidence rates of COVID-19 than women [7]. The gender rates may vary from city to city and from country to country and between regions within a country [8]. The present study showed that people in the age group of 26-40 years old were predominantly affected by COVID-19 (55.7%) compared to other age groups. The mortality rate may increase with the increase in age, particularly in those aged more than 30 years [9,10]; children are also affected by this condition [11,12]. Regarding the occupation of the patients, most of the patients were employed (48.9%) and got infected through direct contact (78.7%) with other infected people; unemployed patients accounted for 24.7% of the cohort. It has been reported that infections could be highly related to employment such as taxi drivers, salespersons, tour guides, and housekeepers and cleaners [19]. Moreover, those who are employed are at a higher risk of infection because of frequent contact with each other and are more likely to be infected through exposure to contaminated surfaces than direct contact with COVID-19 patients [20].

We observed that most of the infected people (93.7%) were symptomatic while the asymptomatic comprised only 6.3% of the entire study cohort. In another study conducted in Saudi Arabia, it was reported that out of 82 cases of COVID-19, 37 (45.1%) were found to be symptomatic and 45 (54.9%) were asymptomatic [21]. In our study, It was found that the majority of the patients (96.6%) had informed their relatives and friends about their disease. It is well-known that telehealth consultation has been availed by many patients during the ongoing COVID-19 pandemic, and relatives and friends can often be relied upon to arrange this. Also, the present study has revealed that 96.6% of patients who were suspected to be at high risk of infection because of typical respiratory infection symptoms were found to be positive for SARS-CoV-2 by reverse transcription-polymerase chain reaction (RT-PCR). In similar epidemiological studies, the positivity rates for SARS-CoV-2 ranged between 5.1 and 14.3% [22,23], which is in contrast with our study, which found that the majority of the tested cases were positive for SARS-CoV-2. For every patient admitted to the hospital, an RT-PCR test was periodically being done.

The study showed that 70.7% of the infected people underwent treatment after admission, and the treatment was performed on the basis of age, comorbidities, respiratory rate, and oxygen saturation. The most important elements of treatment in severe cases of COVID-19 are adequate oxygenation, pharmaceutical prevention of thrombosis, and administration of dexamethasone [24,25]. About 55.2% of patients were not psychologically affected by COVID-19 while 44.2% reported being affected. In one study, 39.1% of the studied participants had psychological distress, indicating that when faced with the COVID-19 epidemic, people were often worried about the risks of infection and protective measures, resulting in psychological distress [26]. Our study also showed that 6.9% of the patients did not follow any precautionary measures. Precautions against COVID-19 include the use of face masks, social distancing, and limiting the number of staff visits. These precautions may significantly lower the incidence of COVID-19 infections [27]. The present study showed that 47.7% of COVID-19 patients were infected through contact with family members. The family members can easily infect each other. One study found that asymptomatic patients can still infect others as asymptomatic patients are able to spread the disease to large numbers of people [28,29]. This study has a major limitation: we could have assessed more variables and parameters related to COVID-19 by reviewing the medical records of the study population, which would have enhanced the validity and strength of our findings.

## Conclusions

The COVID-19 disease continues to spread across the Al-Leith town. The study concluded that people of diverse age groups were susceptible to COVID-19, and direct contact with infected relatives and friends was the main mode of transmission. We also found that a considerable number of infected people did not adhere to precautionary measures, such as using masks, maintaining physical distance, and ensuring non-contact with people in isolation due to infection. A majority of the participants showed symptoms of COVID-19. There is a need for further studies to explore more variables associated with COVID-19, as it is a rapidly spreading and evolving disease, and information about the condition is still scarce.
